# Volume markers in left ventricular diastolic dysfunction and adverse outcomes in peritoneal dialysis patients: a prospective cohort study

**DOI:** 10.1038/s41598-023-43442-x

**Published:** 2023-10-06

**Authors:** Shoubo Xiang, Mingliang Zuo, Yan Deng, Xian Luo, Qianhua Dong, Jin Chen, Chung-Wah Siu, Lixue Yin

**Affiliations:** 1 Department of Cardiology, West China Hospital, Sichuan University, Chengdu, China; 2 Ultrasound in Cardiac Electrophysiology and Biomechanics Key Laboratory of Sichuan Province, Sichuan Provincial People’s Hospital, University of Electronic Science and Technology of China, Chengdu, China; 3 Department of Nephrology, Institute of Nephrology, Sichuan Provincial People’s Hospital, University of Electronic Science and Technology of China, Chengdu, Sichuan China; 4 Cardiology Division, Department of Medicine, Queen Mary Hospital, The University of Hong Kong, Hong Kong SAR, China

**Keywords:** Cardiology, Nephrology

## Abstract

Left ventricular diastolic dysfunction (LVDD) is an early event associated with cardiovascular complications and poor prognosis in chronic kidney disease patients undergoing dialysis. In this study, we investigated whether diastolic dysfunction, measured by the E/E′ ratio, affects adverse outcomes in peritoneal dialysis (PD) patients (n = 148). Our results showed that patients with an E/E′ ratio ≥ 15 were more likely to be female, have a longer dialysis vintage, have significantly higher left atrial volume index and left atrial kinetic energy levels, have lower E′ levels and LV hypertrophy (LVH) degree, and have higher volume markers. Kaplan–Meier curves revealed that patients with a higher E/E′ ratio had worse survival and a higher risk of heart failure than those with a lower E/E′ ratio. Subgroup analysis demonstrated that non-diabetic patients with a higher E/E′ ratio had a higher risk of heart failure than those with a lower E/E′ ratio. Cox proportional hazard regression analysis indicated that the ECW/ICW ratio was strongly associated with LVDD and confirmed that the E/E′ ratio was an independent risk factor for overall death. Our study suggests that monitoring the E/E′ ratio in PD patients is important for improving their prognosis.

## Introduction

Peritoneal dialysis (PD) is an irreplaceable renal replacement therapy for patients with end-stage kidney disease. It is available in 75% of countries and covers approximately 11% of long-term dialysis patients^1^. Cardiovascular disease (CVD) is common among patients undergoing PD and hemodialysis (HD) and is considered the leading cause of death^2^. According to a recent meta-analysis, patients with PD have a higher cardiovascular mortality rate than those with HD. Furthermore, a reported 50% of PD deaths are caused by CVD^3^. Therefore, identifying the risk factors for these events is of great interest.

Cardiac remodeling occurs in dialysis patients before and after dialysis^4^. Although heart failure is often associated with various comorbidities, decreased renal function is particularly important in the treatment of heart failure^5^. Left ventricular diastolic dysfunction (LVDD), a sign of LV pressure overload, is common among patients with end-stage renal disease^6^. Evidence to date has indicated that LVDD occurs earlier than systolic dysfunction in PD patients and that LVDD impairment is the primary determinant of CVD symptoms in this patient population^7^. LVDD can predict the rapid decline in residual renal function and future mortality in PD patients^7,8^. As a result, the early detection of diastolic dysfunction in patients with PD is of great clinical importance and is vital to their treatment.

It is well known that echocardiography is the most direct and effective means of assessing systolic and diastolic dysfunction, in particular, the assessment of LV mass, LV ejection fraction (LVEF), transmission velocity, and the ratio of early diastolic mitral inflow velocity to early diastolic mitral annulus velocity (E/E′)^9^. Several echocardiographic parameters are associated with adverse outcomes in patients with PD^10^. For example, early LV mass index progression was independently linked to all-cause mortality and CVD events in patients with PD. Additionally, a low LVEF increases a patient’s risk of mortality and adverse CVD outcomes compared to individuals without heart failure^11^. While some young PD patients with preserved LVEF already have diastolic dysfunction^12^, this suggests that LVEF may not be as sensitive as once thought at reflecting diastolic dysfunction at an early stage in the disease process.

The E/E′ has been shown to be a useful marker of left ventricular (LV) filling pressures, and has been shown to correlate with left atrial (LA) pressure^13^. Based on guidelines from the American Society of Echocardiography and the European Association of Cardiovascular Imaging, a value of E/E′ > 14 suggests the presence of diastolic dysfunction^14^. Previously, the E/E′ ratio has also been identified as an effective predictor of primary cardiac events in individuals with hypertension^15^. Recent studies demonstrated that the E/E′ ratio, another important parameter of diastolic dysfunction, has strong predictive value for mortality and CVD in patients on dialysis for chronic kidney disease (CKD)^16^, but our understanding of its prognostic value and clinical outcomes in PD patients remains low. This study aimed to investigate the association between E/E′ ratio and adverse outcomes in patients with PD.

## Results

### Baseline clinical characteristics

A total of 148 patients with PD were included in the analysis. The median patient age was 59.9 years, and 65% of them were male. 76% of the population had hypertension and 47% had diabetes mellitus. The mean dialysis vintage was 2.8 ± 4.4 years. A summary of the patient’s baseline characteristics is shown in Table 1. Patients were divided into survival and non-survival groups based on their overall survival. Patients in the non-survival group were older, more likely to have CVD, and had a longer dialysis duration. There were significant differences in the E/E′ ratio, E′, pulmonary capillary wedge pressure, LVEF, fractional shortening, and maximum left anterior volume index (LAVI), while no significant differences were observed in medication, dry body weight, or other parameters. The E/E′ ratio differed significantly between the survival and non-survival groups. Based on an E/E′ ratio of 15^17^, patients were divided into with or without LV diastolic dysfunction categories. Patients with an E/E′ ratio > 15 were more likely to be female, have a longer dialysis vintage, and have significantly higher LAVI and LA kinetic energy levels and lower E′ level and LVH degree. In addition, the volume markers (ECW/ICW and OH/ECW) were significantly higher in the high E/E′ group, whereas the hemoglobin and albumin levels were similar. We have also observed that there is a greater use of ACEI/ARB in PD patients who have an E/E′ ratio of less than 15 (Table 2).

**Table1:** Baseline characteristics for PD patients stratified by death.

	Total (n = 148)	Survival (n = 109)	Non-survival (n = 39)	P value
Age (yr)	59.9 ± 13.3	58.3 ± 13.1	64.7 ± 12.9	0.01
Male (%)	96 (64.8)	69 (63.3)	27 (69.2)	0.52
BMI	23.3 ± 3.5	23.3 ± 3.4	23.2 ± 3.7	0.84
Dry body weight	59.5 ± 11.3	59.9 ± 10.9	58.6 ± 12.6	0.56
Systolic BP, mmHg	141.8 ± 24.3	140.0 ± 23.3	148.0 ± 25.8	0.08
Diastolic BP, mmHg	77.8 ± 12.6	76.9 ± 12.5	80.6 ± 12.3	0.13
Co-existing comorbidities, no. (%)				
CVD, no. (%)	29(19.6)	19 (17.4)	10 (25.6)	0.023
Diabetes, no. (%)	69 (46.6)	51 (46.8)	18 (46.1)	0.92
Hypertension, no. (%)	112 (75.7)	82 (75.2)	30 (76.9)	0.87
Smoking, no. (%)	35 (23.6)	24(22.0)	11 (28.2)	0.41
Dialysis vintage, years	2.8 ± 4.4	1.9 ± 3.5	5.4 ± 5.4	0.00
ECW/ICW ratio	0.94 ± 0.16	0.92 ± 0.15	0.98 ± 0.17	0.08
OH/ECW ratio	0.12 ± 0.10	0.11 ± 0.10	0.14 ± 0.10	0.25
Echocardiography				
LVMI, g/m^2^	160.9 ± 64.7	160.4 ± 63.5	165.5 ± 67.2	0.69
E/E′ ratio	14.7 ± 7.5	13.3 ± 4.9	18.7 ± 11.5	0.00
E′, cm/s	5.8 ± 2.2	6.1 ± 2.4	4.9 ± 1.8	0.001
PCWP, mmHg	21.1 ± 9.8	19.3 ± 6.4	26.4 ± 14.9	0.00
Kinetic energy, kdynes, cm/m^2^	9.6 ± 8.2	9.6 ± 7.8	9.8 ± 9.2	0.87
PASP, mmHg	30.1 ± 10.3	29.2 ± 8.5	32.0 ± 13.0	0.24
LVEF (%)	67.3 ± 9.4	68.8 ± 8.5	63.0 ± 10.7	0.001
FS (%)	38.5 ± 7.8	39.6 ± 7.6	35.5 ± 7.9	0.008
Relative wall thickness	0.54 ± 0.26	0.52 ± 0.22	0.59 ± 0.39	0.09
LAD, mm	38.5 ± 33.5	38.2 ± 31.1	39.2 ± 40.2	0.09
LAVI_max_, ml/m^2^	37.0 ± 14.6	34.9 ± 13.9	42.9 ± 16.5	0.005
LVH, n(%)	99 (66.9)	73 (66.9)	26 (66.7)	0.94
Hypertrophy (Concentric/ eccentric, n)	58/32	43/24	15/8	0.79
Valve disease, n(%)	45 (30.4)	29 (26.6)	16 (41.0)	0.09
Laboratory parameters				
Hemoglobin (g/dL)	9.8 ± 1.4	9.7 ± 1.5	10.0 ± 1.3	0.23
Alkaline phosphatase, U/L	100.8 ± 70.1	104.1 ± 78.6	91.5 ± 46.3	0.32
Serum albumin (g/L)	36.6 ± 3.9	36.7 ± 4.0	36.4 ± 3.7	0.73
Calcium*phosphorus	3.9 ± 1.1	3.9 ± 1.2	3.8 ± 0.9	0.67
High sensitive c-reactive protein (mg/L)	0.7 ± 0.6	0.6 ± 0.6	0.8 ± 0.6	0.63
Phosphorus	1.6 ± 0.4	1.6 ± 0.4	1.6 ± 0.4	0.95
iPTH, pg/L	104.9 ± 174.3	101.7 ± 152.3	113.9 ± 235.9	0.72
BNP, pg/mL	116.8 ± 141.9	113.9 ± 142.8	125.1 ± 139.3	0.32
Medication				
Statins, n(%)	68 (45.9)	49 (44.9)	19 (48.7)	0.71
Antiplatelets, n(%)	43 (29.1)	29 (26.6)	14 (35.9)	0.29
ACEI/ARB, n(%)	108 (72.9)	81 (74.3)	27 (69.2)	0.58
EPO injection, n(%)	88 (59.5)	61 (55.9)	27 (69.2)	0.19

*BMI* body mass index, *CVD* cardiovascular disease, *LVMI* left ventricular mass index, *PCWP* pulmonary capillary wedge pressure, *PASP* pulmonary artery systolic pressure, *LVEF* left ventricular ejection fractions, *FS* fractional shortening, *LAD* left atrium diameter, *LVH* left ventricular hypertrophy, *iPTH* Intact parathyroid hormone, *BNP* brain natriuretic peptide, *ACEI* angiotensin-converting enzyme (ACE) inhibitors, *ARB* angiotensin receptor block, *EPO* erythropoietin.

**Table 2:** Baseline characteristics of the study population according to E/E′ ratio at baseline.

	E/E′ ratio < 15 (n = 96)	E/E′ ratio ≥ 15 (n = 52)	P value
Demographic data			
Age, years	59.7 ± 13.7	60.4 ± 12.8	0.84
Male, n(%)	70 (72.9)	26 (50.0)	0.016
BMI, kg/m^2^	23.0 ± 3.59	23.6 ± 2.9	0.27
Dry body weight	59.6 ± 12.0	59.3 ± 9.9	0.89
Systolic BP, mmHg	139.6 ± 24.1	147.7 ± 24.4	0.06
Diastolic BP, mmHg	78.0 ± 12.7	77.2 ± 13.2	0.75
Co-existing comorbidities, no.(%)			
CVD, no. (%)	18 (18.7)	11(21.1)	0.32
Diabetes, no. (%)	40 (41.7)	29 (55.8)	0.06
Hypertension, no. (%)	69 (71.9)	43 (82.7)	0.14
Smoking, no. (%)	26 (27.1)	9 (17.3)	0.08
Dialysis vintage, years	2.1 ± 4.3	4.0 ± 4.1	0.015
ECW/ICW ratio	0.91 ± 0.17	0.99 ± 0.13	0.002
OH/ECW ratio	0.10 ± 0.10	0.15 ± 0.09	0.04
Echocardiography			
LVMI, g/m^2^	153.8 ± 64.4	174.8 ± 64.1	0.07
E’, cm/s	6.4 ± 2.2	4.4 ± 1.2	0.00
PCWP, mmHg	16.3 ± 3.1	30.2 ± 11.5	0.00
LA Kinetic energy, kdynes, cm/m^2^	8.3 ± 8.0	11.9 ± 8.5	0.015
PASP, mmHg	28.9 ± 9.3	30.8 ± 12.1	0.39
LVEF, %	67.5 ± 9.2	66.9 ± 10.1	0.68
FS, %	39.0 ± 6.6	37.7 ± 9.7	0.38
Relative wall thickness	0.51 ± 0.11	0.61 ± 0.42	0.12
LAD, mm	38.1 ± 32.0	39.2 ± 36.2	0.71
LAVImax, ml/m^2^	34.9 ± 12.9	40.9 ± 17.9	0.02
LVH, n(%)	60 (62.5)	39 (75.0)	0.08
Hypertrophy (Concentric/ eccentric, n)	34/20	24/12	0.37
Valve disease, n(%)	30 (31.2)	15 (28.8)	0.67
Laboratory parameters			
Hemoglobin, g/dL	9.9 ± 1.5	9.6 ± 1.3	0.68
Alkaline phosphatase, U/L	103.4 ± 83.7	95.9 ± 44.9	0.46
Serum albumin, g/L	36.5 ± 4.1	36.8 ± 3.5	0.90
Calcium*phosphorus	3.9 ± 1.2	3.9 ± 0.9	0.88
High-sensitive c-reactive protein, mg/L	0.6 ± 0.5	0.7 ± 0.7	0.24
Phosphorus, mg/dL	1.6 ± 0.4	1.6 ± 0.3	0.88
iPTH, pg/L	115.1 ± 200.1	86.1 ± 126.6	0.17
BNP, pg/mL	110.6 ± 144.7	134.2 ± 133.9	0.06
Medication			
Statins, n(%)	44(45.8)	24(46.2)	0.92
Antiplatelets, n(%)	27(28.1)	16(30.8)	0.78
ACEI/ARB, n(%)	72(75.0)	36(69.2)	0.48
EPO injection, n(%)	53(55.2)	35(67.3)	0.17
Heart failure, n(%)	6 (6.3)	15(28.8)	0.000
CV mortality, n(%)	7 (7.3)	5 (9.6)	0.65
All-cause death, n(%)	19(19.8)	20 (38.5)	0.017

*BMI* body mass index, *CVD* cardiovascular disease, *LVMI* left ventricular mass index, *PCWP* pulmonary capillary wedge pressure, *PASP* pulmonary artery systolic pressure, *LVEF* left ventricular ejection fractions, *FS* fractional shortening, *LAD* left atrium diameter, *LVH* left ventricular hypertrophy, *iPTH* Intact parathyroid hormone, *BNP* brain natriuretic peptide, *ACEI* angiotensin-converting enzyme (ACE) inhibitors, *ARB* angiotensin receptor block, *EPO* erythropoietin.

### Adverse events according to E/E′ ratio

**Figure 1:**
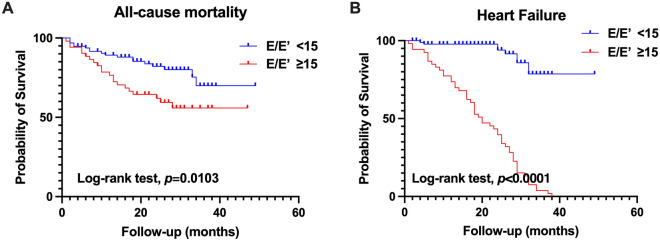
Kaplan–Meier survival curves for (**A**) all-cause mortality and (**B**) heart failure according to LV diastolic dysfunction. LV diastolic dysfunction was defined as E/E′ ratio > 15.

**Figure 2:**
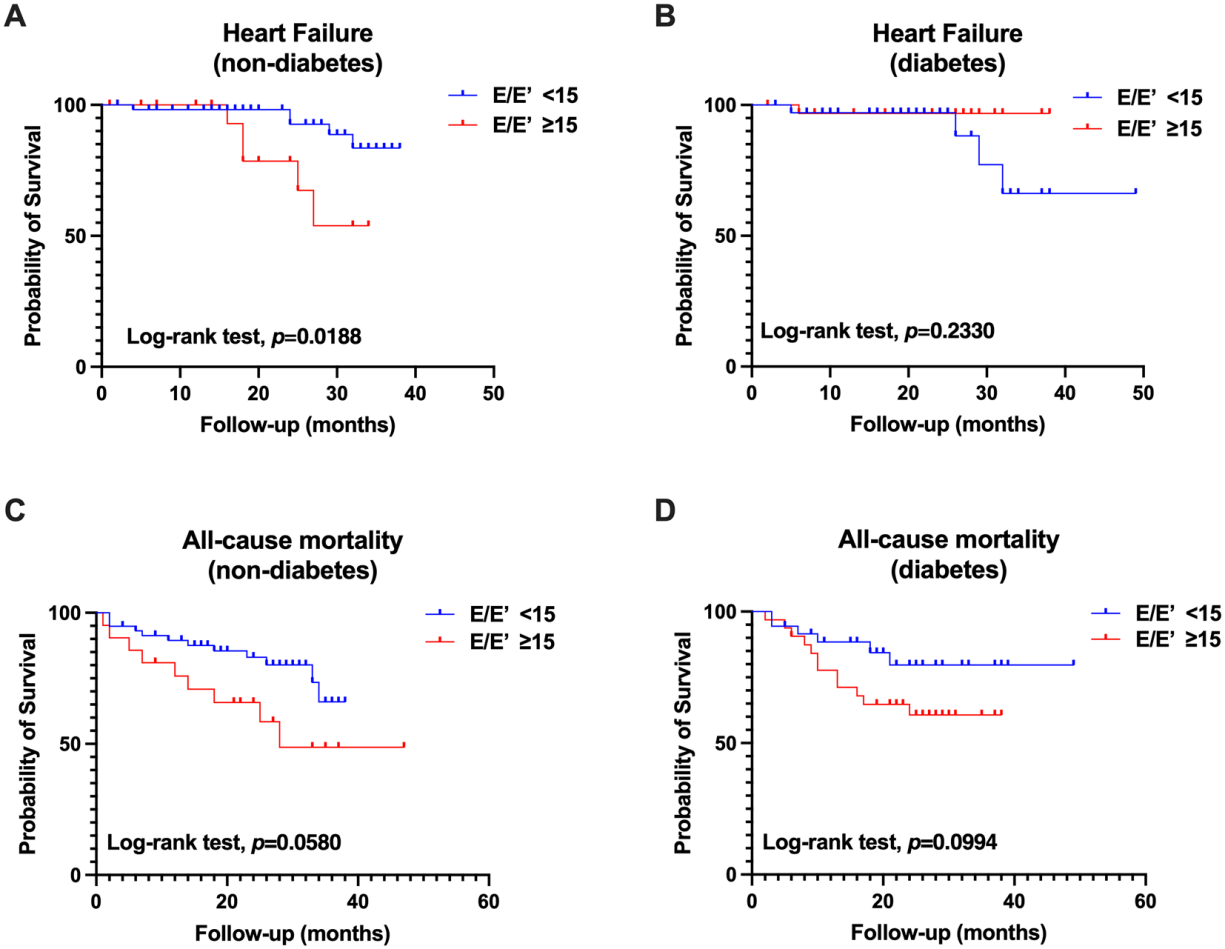
Kaplan–Meier survival curves for heart failure and overall death according to LV diastolic dysfunction stratified by diabetes. (**A,C**) non-diabetes; (**B,D**) diabetes; LV diastolic dysfunction was defined as E/E′ ratio ≥ 15.

**Figure 3:**
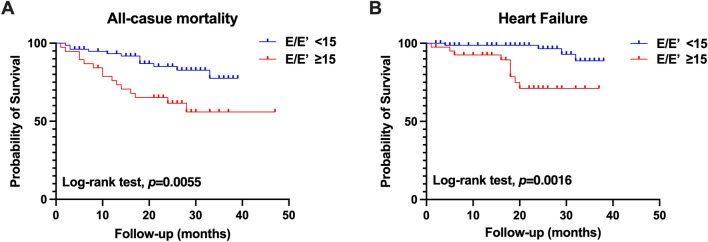
Kaplan–Meier survival curves for (**A**) all-cause mortality and (**B**) heart failure according to LV diastolic dysfunction for peritoneal dialysis patients excluded prior cardiovascular events. LV diastolic dysfunction was defined as E/E′ ratio   ≥  15.

During a median follow-up of 21.8 ± 10.8 months, 39 patients (26.4%) died. The cause of death was attributed to issues with the cardiovascular system (CVS) (30.8%), infections (20.5%), and other factors (48.7%). Notably, there were no significant differences in the cause of death between the group with an E/E′ ratio < 15 and the group with an E/E′ ratio ≥ 15 (Supplementary Fig. 1). Patients with higher E/E′ ratios had a significantly higher risk of all-cause mortality (19.8% vs. 38.5%, p = 0.017). Kaplan–Meier curves showed that patients with a higher E/E′ ratio had a worse survival rate than those with a lower E/E′ ratio (p = 0.0103; Fig. 1A). Moreover, the incidence of heart failure was significantly different between the low and high E/E′ ratio groups (p < 0.0001; Fig. 1B). Moreover, the subgroup analysis showed that those without diabetes with higher E/E′ ratios had a higher risk of heart failure than those with lower E/E′ ratios (p = 0.0188; Fig. 2A,B), while not for overall death (Fig. 2C,D). Additionally, we performed Kaplan–Meier survival analysis for PD patients without CVD history, and the results also indicated that higher E/E′ ratios increased the risk of all-cause mortality and heart failure (Fig. 3A,B).To adjust for confounding effects, we conducted uni- and multivariate Cox proportional hazard regression analyses (Table 3). Age (hazard ratio [HR], 1.05; 95% confidence interval [CI], 1.03–1.10; p = 0.039), E/E′ ratio (HR, 1.1; 95% CI, 1.1–1.2; p = 0.047) and LAVI (HR, 1.1; 95% CI, 1.0–1.1; p = 0.038) were associated with all-cause mortality.

**Table 3:** Univariate and multivariate Cox-regression analysis for predicting all-cause mortality in PD patients (n = 148).

Covariates	Univariate		Multivariate	
	Hazard ratio (95CI)	P value	Hazard ratio (95%CI)	P value
Age	1.1 (1.0–1.1)	0.009	1.05(1.03–1.10)	0.039
Gender	1.3 (0.6–2.5)	0.51		
BMI	0.9 (0.8–1.1)	0.84		
Dry body weight	0.9 (0.9–1.0)	0.53		
Dialysis vintage	1.11 (1.1–1.2)	0.00	0.95	0.43
Systolic BP, mmHg	1.0 (0.9–1.1)	0.10	1.02	0.15
Diastolic BP, mmHg	1.0(0.9–1.1)	0.17		
CVD	1.3 (0.6–2.9)	0.76		
Diabetes	1.0 (0.5–1.9)	0.97		
Hypertension	1.0 (0.4–2.3)	0.99		
Smoking	1.6 (0.7–3.5)	0.41		
ECW/ICW ratio	1.2 (1.0–1.3)	0.01	1.1(1.1–1.2)	0.35
OH/ECW	3.1 (0.3–14.6)	0.27		
Echocardiography				
LVMI	1.0(0.9–1.1)	0.49		
E/E′ ratio	1.1 (1.0–1.1)	0.001	1.1 (1.1–1.2)	0.047
PASP	1.0 (0.9–1.1)	0.26		
LVEF	0.9 (0.8–0.9)	0.003	1.0(0.9–1.1)	0.07
FS	0.9 (0.8–0.9)	0.008		
LAVImax	1.1(1.0–1.1)	0.004	1.1 (1.0–1.1)	0.038
Laboratory parameters				
Hemoglobin	1.2 (1.0–1.5)	0.08		
Alkaline phosphatase	1.0 (1.0–1.1)	0.34		
Serum albumin	1.0 (0.9–1.1)	0.99		
Calcium*phosphorus	0.9 (0.7–1.2)	0.56		
High-sensitive c-reactive protein	1.1 (0.7–1.9)	0.58		
BNP	1.1 (1.1–1.2)	0.07		
Statins	1.1(0.6–2.1)	0.67		
Antiplatelets	1.7(0.8–3.2)	0.13		
ACEI/ARB	0.9(0.5–1.8)	0.83		
EPO injection	1.5(0.8–3.1)	0.16		

*BMI* body mass index, *CVD* cardiovascular disease, *LVMI* left ventricular mass index, *PCWP* pulmonary capillary wedge pressure, PASP pulmonary artery systolic pressure, *LVEF* left ventricular ejection fractions, *FS* fractional shortening, *LAD* left atrium diameter, *LVH* left ventricular hypertrophy, *iPTH* Intact parathyroid hormone, *BNP* brain natriuretic peptide, *ACEI* angiotensin-converting enzyme (ACE) inhibitors, *ARB* angiotensin receptor block, *EPO* erythropoietin.

### Correlation and risk factors for LVDD

**Figure 4:**
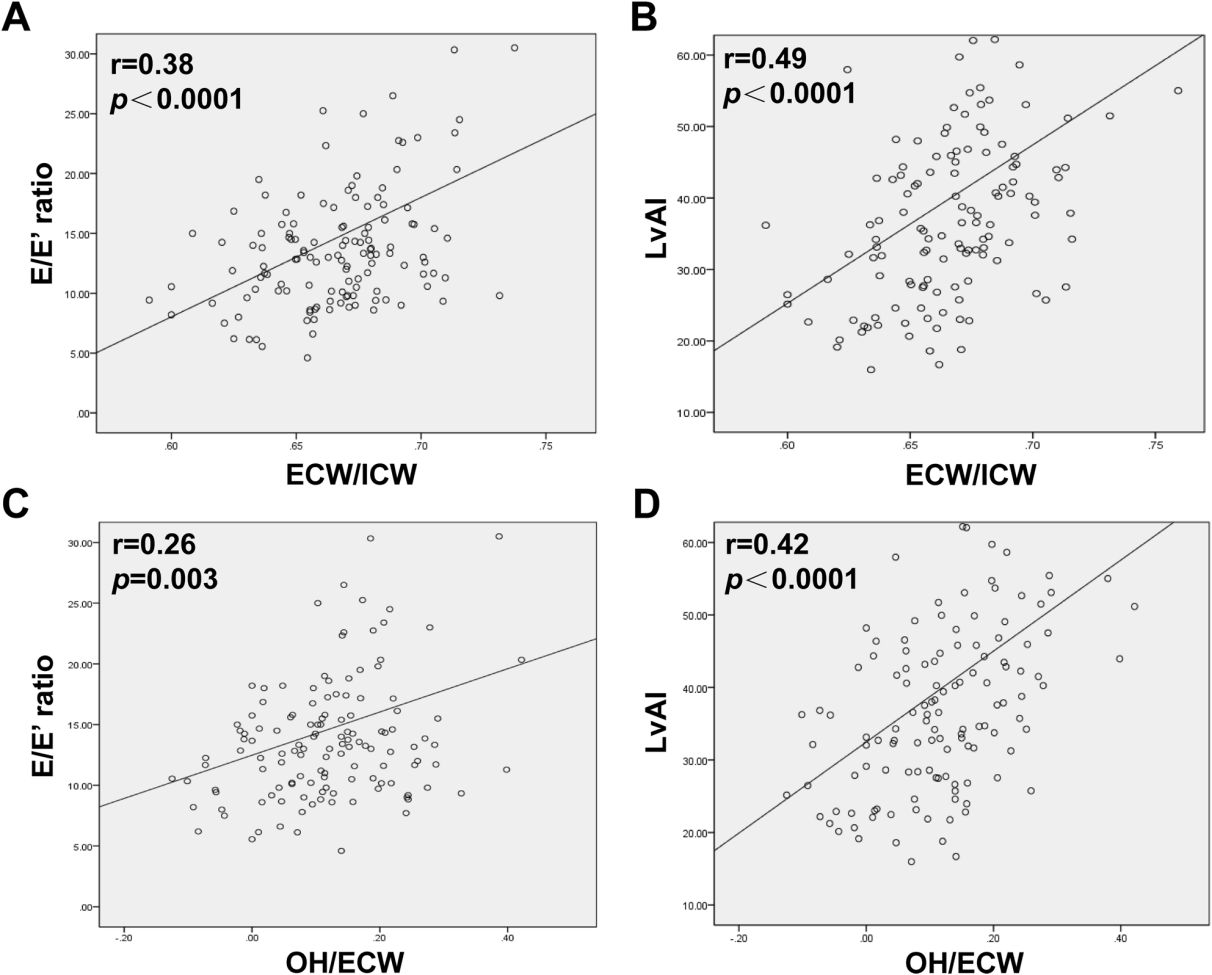
The correlation between LV diastolic dysfunction and fluid overload. (**A**) ECW/ICW had a positive correlation with E/E′ ratio (r = 0.38, p < 0.0001); (**B**) ECW/ICW had a positive correlation with LVAI (r = 0.49, p < 0.0001); (**C**) OH/ECW had a positive correlation with E/E′ (r = 0.26, p = 0.003); (**D**) OH/ECW had a positive correlation with LVAI (r = 0.42, p < 0.0001).

Uni- and multivariate logistic regression analyses were conducted to investigate the risk factors associated with LVDD. As shown in Table 4, there was a correlation between volume markers and LVDD. Interestingly, the ECW/ICW ratio represents the volume status, with a significant positive correlation between the E/E′ ratio (r = 0.38, p < 0.0001; Fig. 4A) and LVAI (r = 0.49, p < 0.0001; Fig. 4B). Left atrial size is also a surrogate marker for chronically augmented LV diastolic pressure^18^, and an LAVI > 34 mL/m^2^ predicted death^19^. Relative overhydration (OH/ECW) showed a significant positive correlation with E/E′ ratio (r = 0.26, p = 0.003; Fig. 4C) and LVAI (r = 0.42, p < 0.0001; Fig. 4D). However, the multivariate logistic regression analysis results revealed that only ECW/ICW (HR, 1.18; 95% CI, 1.06–1.34; p = 0.027; Table 4) was a significant independent risk factor for LVDD in the multivariate logistic regression analysis.

**Table 4:** Univariate and multivariate logistic regression analysis for LV diastolic dysfunction.

Covariates	Univariate		Multivariate	
	Hazard ratio (95%CI)	P value	Hazard ratio (95%CI)	P value
Age, years	1.03	0.84		
Male	2.45	0.017	0.55 (0.25–1.19)	0.13
Smoking status	0.55 (0.18–1.61)	0.25		
Diabetes	1.95 (0.95–3.99)	0.06		
BMI	1.06 (0.96–1.17)	0.27		
Dry body weight	0.98 (0.92–1.02)	0.88		
CVD	0.85 (0.32–2.28)	0.25		
Hypertension	2.16 (0.76–6.11)	0.14		
Systolic BP	1.01 (1.00–1.03)	0.07		
Diastolic BP	0.99 (0.97–1.02)	0.75		
Dialysis vintage, years	1.11 (1.04–1.21)	0.02	1.08 (1.00–1.18)	0.06
ECW/ICW	1.23 (1.07–1.41)	0.004	1.18 (1.06–1.34)	0.027
OH/ECW	37.55 (1.14–1237)	0.04		
Hemoglobin	0.95 (0.75–1.21)	0.67		
Alkaline phosphatase	0.99 (0.99–1.00)	0.47		
Serum albumin	0.99 (0.91–1.09)	0.90		
Calcium*phosphorus	1.02 (0.75–1.39)	0.88		
High-sensitive c-reactive protein	1.47 (0.82–2.64)	0.19		
Total cholesterol	0.92 (0.70–1.21)	0.56		
iPTH	0.99 (0.99–1.00)	0.33		
BNP	1.12 (0.98–1.19)	0.10		
Statins	0.91 (0.42–1.81)	0.75		
Antiplatelets	0.84 (0.44–1.82)	0.61		
ACEI/ARB	1.41 (0.73–3.1)	0.35		
EPO injection	0.75 (0.36–1.42)	0.29		

*BMI* body mass index, *CVD* cardiovascular disease, *LVMI* left ventricular mass index, *PCWP* pulmonary capillary wedge pressure, *PASP* pulmonary artery systolic pressure, *LVEF* left ventricular ejection fractions, *FS* fractional shortening, *LAD* left atrium diameter, *LVH* left ventricular hypertrophy, *iPTH* Intact parathyroid hormone, *BNP* brain natriuretic peptide, *ACEI* angiotensin-converting enzyme (ACE) inhibitors, *ARB* angiotensin receptor block, *EPO* erythropoietin.

## Discussion

This study revealed that E/E′ ratio could represent an independent risk factor for heart failure and overall mortality in patients with PD. Furthermore, it was positively correlated with volume overload.

In clinical settings, E/E′ ratio can be measured using ultrasonic Doppler, which assesses the systolic and diastolic functions of the heart. In particular, the E/E′ ratio plays a crucial role in determining cardiac diastolic function. In a national echocardiography registry, diastolic dysfunction was strongly associated with heart disease–related mortality^20^. When the E/E′ ratio is < 8, cardiac diastolic insufficiency can generally be ruled out. In contrast, an E/E′ ratio > 15 indicates diastolic dysfunction, suggesting impaired ventricular relaxation^21^. Among the CKD population, the average E/E′ ratio was approximately 9.6, while when the estimated glomerular filtration rate was < 45 mL/min, the ratio increased to 10.1. More importantly, a low estimated glomerular filtration rate was an independent risk factor of an elevated E/E′ ratio (> 15)^22^. In addition, the E/E′ ratio was positively correlated with hypertension^23^. A continuous decrease in the E/E′ ratio was observed in patients undergoing dialysis. Nevertheless, a previous study showed no significant difference in E/E′ between patients on HD versus PD^24^. A study of patients with PD followed them for 3 years to determine the dynamic changes in LV structure and function. In their study, only distal function parameters were statistically decreased, including the E/A ratio and e′, while the E/E′ ratio increased^25^, indicating that PD patients with long-term structural and functional changes showed nonparallel progression and distal function may be more affected. We observed that > 35% of PD patients had an E/E′ ratio > 15, suggesting that LVDD is a very common complication in patients with PD. Moreover, patients with a higher E/E′ ratio had a relatively long dialysis duration, indicating that LVDD is closely related to dialysis vintage. Previous reports have also indicated a positive correlation between dialysis vintage and LVEF^26^. The study results indicate that dialysis duration has a significant impact on cardiac function in PD patients. However, the E/E′ ratio was reportedly significantly decreased in pediatric PD patients after renal transplantation^27^, strongly suggesting that LVDD could be partially reversed while renal function improves. Therefore, PD treatment might involve the routine evaluation of diastolic function and E/E′ ratio levels.

As the E/E′ ratio is the best indicator of cardiac diastolic function, previous studies demonstrated that the E/E′ ratio is more sensitive than the E/A ratio for detecting LVDD in patients with systemic sclerosis^28^. Furthermore, E/E′ ratio is a powerful predictor of primary cardiac events in a hypertensive population^29^. During low-level exercise, the E/E′ ratio can be applied in the diagnosis of heart failure with preserved LVEF^30^. The E/E′ ratio can predict mortality and CVD in patients with CKD disease and diastolic dysfunction^31^. Moreover, it is also a sensitive indicator of diastolic dysfunction in PD patients^30^. Exercise-induced elevated E/E′ ratios may be a reliable indicator of CV events in patients with end-stage renal disease undergoing continuous ambulatory PD^32^. Our study showed for the first time that the E/E′ ratio is associated with an increased risk of all-cause mortality. We also summarized the E/E′ ratio and adverse outcomes, which supports the important role of E/E′ ratio in CKD (Supplementary Table 1). Furthermore, we also found that the E/E′ ratio was associated with an increased risk of heart failure, while a subgroup analysis found that non-diabetic patients with high E/E′ ratios had higher heart failure rates than those with low E/E′ ratios, which warrants further investigation.

Fluid overload was defined as ECW/total body water assessed using BIA. Fluid management is crucial for reducing cardiovascular risk in patients with PD. BIA has become widely used for assessing the volume status of dialysis patients^33^. With BIA-guided fluid management, PD patients with fluid overload were significantly less likely to suffer from fluid overload than those with traditional methods^34^. A previous study showed that the left atrial diameter, E/E′ ratio, and LVEF were significantly lower in overhydrated than normohydrated patients^35^. Moreover, the E/E′ ratio showed a positive correlation with brain natriuretic peptide^36^ as well as fluid overload in predialysis CKD patients^37^. The mechanism underlying LVDD is complex. Overhydration may contribute to LVDD by increasing LV preload. Because of excessive sodium loading, volume overload usually results in cardiac dilation and increased LV mass, resulting in LVDD. Anemia, inflammation, and mineral disorders are also thought to play a role in LVDD development, in addition to LVH and neurohumoral alterations (renal-angiotensin-aldosterone system activation)^38^. During the course of our study, we discovered a significant prevalence of anemia among the population under examination. Further analysis revealed that this anemia could potentially be attributed to the underutilization of erythropoietin (EPO). We also observed that injections of EPO may serve as a protective factor against LV diastolic dysfunction. Our study also demonstrated that PD patients with a higher E/E′ ratio had higher ECW/ICW and OH/ECW ratios. This finding suggests that volume control has a beneficial effect on cardiac function.

However, some limitations of this study require consideration. In this single-center retrospective study, BIS and echocardiography were performed only once at the beginning of PD, and no relationship was established between the dynamics of these indicators and prognosis. Further research is required to confirm this relationship. In addition to the aforementioned, it should be noted that the lack of information regarding disease-specific causes of death has significant implications for the health management of PD patients. Unfortunately, the causes of over 50% of deaths are attributed to the "unknown", indicating a lack of investigation into the underlying factors leading to these fatalities.

In conclusion, according to our study, diastolic dysfunction, measured by the E/E′ ratio, is associated with fluid overload, heart failure, and overall death, and routine monitoring of these parameters is essential for patients with PD.

## Materials and methods

### Study population

Our study is a single-center retrospective study. We analyzed patients who received continuous ambulatory PD treatment from the Department of Nephrology at Sichuan Provincial People's Hospital between March 2015 and April 2016. The study inclusion criteria were: (1) ≥ 18 years of age and have been receiving PD for more than 3 months with regular follow-up; and (2) undergo four exchanges per day on PD. During the follow-up period, patients who underwent kidney transplantation or hemodialysis were excluded. Participants were required to provide written consent before taking part in the study. The study protocol was approved by the Institutional Review Board of Sichuan Provincial People's Hospital (no. 2022–415) and complied with the declaration of Helsinki.

### Clinical and biochemical parameters

During PD treatment, demographic information such as age, sex, height, weight, PD vintage, smoking status, and comorbidities was obtained from the electronic medical record system(EMRS) of Sichuan Provincial People’s Hospital. Blood samples were taken in the morning after an overnight fast of at least 8 h between 7:00 a.m. and 9:00 a.m. Laboratory measurements, including hemoglobin, serum albumin, high-sensitivity C-reactive protein, alkaline phosphatase, calcium, phosphorus, and intact parathyroid hormone (iPTH), were obtained using the Beckman AU5800 automatic biochemical analyzer, following the manufacturer’s protocol. In addition, we collected data on the usage of medications such as statins, antiplatelets, and ACEI/ARB. CVD is defined as the occurrence of coronary artery disease, arrhythmia, peripheral vascular disease, or cerebral vascular disease.

### Echocardiography examination

We performed transthoracic echocardiography at baseline as described previously. All participants underwent two-dimensional, M-mode, and Doppler imaging using a 3.5-MHz transducer (Vivid 7; GE Vingmed Ultrasound AS, Horten, Norway). The two-dimensional and M-mode echocardiograms were used to measure chamber size, wall thickness, and LVEF. Pulse and tissue Doppler imaging were used to determine the early transmitral flow velocity (E) and early mitral annular velocity (E′), and the E/E′ ratio calculation. The biplane Simpson’s method was used to calculate the left atrial volume (LAV) corrected for body surface area (BSA). LV mass and relative wall thickness were calculated as reported previously^39^, and the former was corrected for BSA. LA kinetic energy and pulmonary capillary wedge pressure were also estimated^40,41^. Pulmonary arterial systolic pressure was assessed by adding the transtricuspid regurgitation gradient to the mean right atrial pressure.

### Volume status assessment

We performed whole-body spectral bioimpedance spectroscopy (BIA) using a Body Composition Monitor (Fresenius Medical Care, Deutschland GmbH) on all participants^42,43^. Based on Cole’s model and equations for body composition spectroscopy, we measured the intracellular to extracellular fluid volume using an emission frequency of 5–1000 kHz. Extracellular water (ECM) and intracellular water (ICW) were calculated. Overhydration (OH) was calculated as the difference between the normal ECW and the actual ECW^44^. Fluid overload is defined as a relative overhydration (OH/ECW) > 15%^45^.

### Endpoint and follow-up

Our primary objectives were to determine the incidence of all-cause death and hospitalization due to worsening heart failure. All-cause mortality is defined as death resulting from any cause, mainly cardiovascular-related mortality. Heart failure is defined as episodes that clearly require hospitalization, according to the Heart Failure Society of America's definition^46^. To achieve this, we manually collected data on these endpoints from the medical records of all the patients enrolled in the study. Our team of experienced nephrologists and cardiologists then adjudicated the data to ensure its accuracy and reliability. We followed up with all participants from the screening date until April 30, 2019, or until their death.

### Statistical analysis

Descriptive data are expressed as mean ± standard deviation, while continuous variables are expressed as median, and categorical variables are expressed as frequencies or percentages. Continuous variables were analyzed using the Mann–Whitney U test. Spearman’s correlation coefficient analysis was used to determine the linear relationship between two continuous variables. Categorical variables were compared using the chi-squared test or Fisher’s exact test as necessary. Pearson’s correlation analysis was used to determine the relationship between volume markers and LVDD. We conducted univariate and multivariate logistic regression analyses to identify independent risk factors for LVDD. For the survival analysis, we used a Kaplan–Meier analysis and log-rank test to compare survival rates between patients with and without LVDD. A Cox proportional hazard regression model was constructed to assess the effects on all-cause mortality. The statistical analysis was performed using IBM SPSS version 22 (Armonk, NY, USA) and GraphPad Prism version 9.5 (GraphPad Software, USA). Statistical significance was set at p < 0.05.

### Statement of Ethics

The participants were required to provide informed written consent prior to participating in the study. The study protocol was approved by the Institutional Review Board of Sichuan Provincial People’s Hospital (no. 2022-415).

### Supplementary Information

Supplementary Information.

## Data Availability

The data are available from the corresponding author on reasonable request.
