# Supplementary material for: Volume markers in left ventricular diastolic dysfunction and adverse outcomes in peritoneal dialysis patients: a prospective cohort study
Source: Sci Rep. 2023 Oct 6;13:16892. doi: 10.1038/s41598-023-43442-x (PMC10558440; doi:10.1038/s41598-023-43442-x)
Supplement: Supplementary file 1 — Supplementary Information. [file 41598_2023_43442_MOESM1_ESM.pdf]

Supplementary Material

Supplementary Figure 1. The causes of death between the E/E' ratio <15 group and the E/E' ratio ≥15 group. CVS: cardiovascular system.

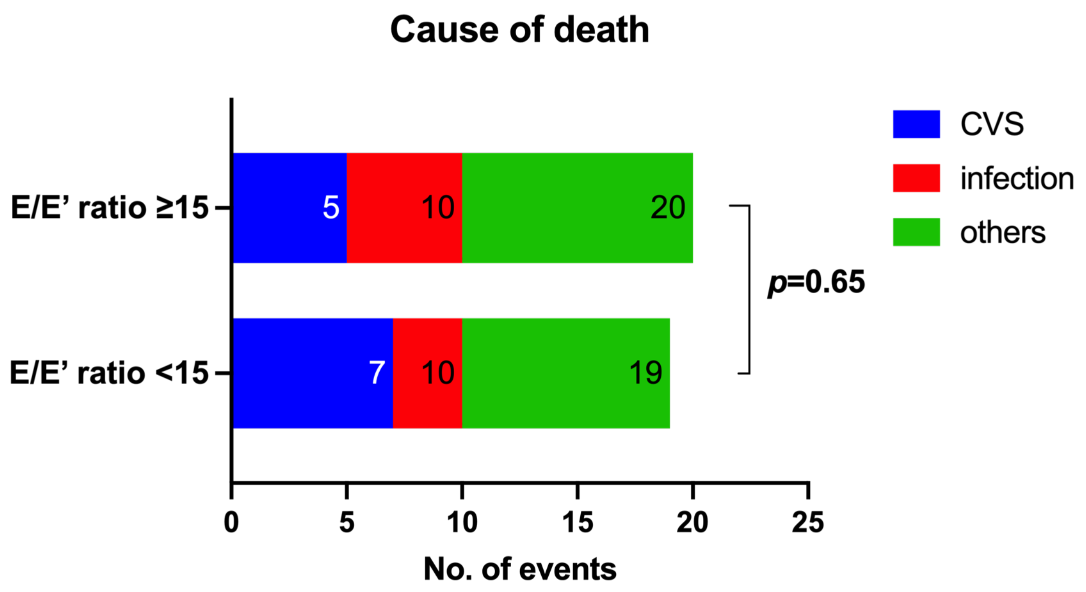

Supplementary Table 1. The E/E' ratio and outcomes in chronic kidney disease

Study	Year	Number	Patients	Outcomes
Kim *et al.* [1]	2012	82	Peritoneal Dialysis	E/E'>15 is associated with RRF decline rate, renal survival and CVD events.
Yoon *et al.*[2]	2013	92	Peritoneal Dialysis	Increased E/E' ratio were independent predictors for mortality and CV events.
Han *et al.* [3]	2015	193	Hemodialysis	E/E' for acute heart failure, and E/e' for all-cause mortality and all combined events.
Kim *et al.* [4]	2016	242	Kidney transplantation	High E/E' ratios were risk factors of major cardiovascular complications
Bang *et al.* [5]	2016	1045	Kidney transplantation	Preoperative E/E' is a prognostic indicator of overall mortality in ESRD patients undergoing KT.
Shin *et al.* [6]	2016	30	Peritoneal Dialysis	Exercise-induced elevated E/e' was significantly associated with CV events
Kim *et al.* [7]	2017	84	Chronic kidney disease	E/E'>15 had significantly higher serum NT-proBNP and overhydration
Huang *et al.* [8]	2019	190	Hemodialysis	The E/E' ratio significantly predicts mortality and CV outcomes and outperforms conventional echocardiographic parameters in outcome prediction in HD patients
Han *et al.* [9]	2019	103	Hemodialysis	E/E' was a significant predictor of hospitalization
Kang *et al.* [10]	2022	2238	Chronic kidney disease	E/E' is associated with renal events in chronic kidney disease
Suh *et al.* [11]	2022	2,135	Chronic kidney disease	LVDD is independently associated with adverse CV outcomes and all-cause mortality in patients with pre-dialysis CKD.

References

1 Kim, J. K. et al. Left ventricular diastolic dysfunction as a predictor of rapid decline of residual renal function in patients with peritoneal dialysis. J Am Soc Echocardiogr 25, 411-420, doi:10.1016/j.echo.2011.11.026 (2012).

2 Yoon, H. E. et al. The prognostic value of abdominal aortic calcification in peritoneal dialysis patients. Int J Med Sci 10, 617-623, doi:10.7150/ijms.5773 (2013).

3 Han, S. S. et al. Predictive value of echocardiographic parameters for clinical events in patients starting hemodialysis. J Korean Med Sci 30, 44-53, doi:10.3346/jkms.2015.30.1.44 (2015).

4 Kim, E. J., Chang, S., Kim, S. Y., Huh, K. H., Kang, S. & Choi, Y. S. Predictive Value of Echocardiographic Abnormalities and the Impact of Diastolic Dysfunction on In-hospital Major Cardiovascular Complications after Living Donor Kidney Transplantation. Int J Med Sci 13, 620-628, doi:10.7150/ijms.15745 (2016).

5 Bang, J. Y. et al. High Left Ventricular Filling Pressure on Doppler Echocardiography Is Associated With Graft Failure and Overall Mortality Following Kidney Transplantation. J Cardiothorac Vasc Anesth 30, 585-591, doi:10.1053/j.jvca.2015.10.006 (2016).

6 Shin, J. H. et al. Treadmill exercise-induced E/e' elevation as a predictor of cardiovascular event in end-stage renal disease on peritoneal dialysis. Korean J Intern Med, doi:10.3904/kjim.2016.254 (2016).

7 Kim, J. S., Yang, J. W., Yoo, J. S., Choi, S. O. & Han, B. G. Association between E/e´ ratio and fluid overload in patients with predialysis chronic kidney disease. PLoS One 12, e0184764, doi:10.1371/journal.pone.0184764 (2017).

8 Huang, W. M., Lin, Y. P., Chen, C. H. & Yu, W. C. Tissue Doppler imaging predicts outcomes in hemodialysis patients with preserved left ventricular function. J Chin Med Assoc 82, 351-355, doi:10.1097/jcma.0000000000000078 (2019).

9 Han, J. H. et al. Diastolic dysfunction is an independent predictor of cardiovascular events in incident dialysis patients with preserved systolic function. PLoS One 10, e0118694, doi:10.1371/journal.pone.0118694 (2015).

10 Kang, E. et al. Left Ventricular Diastolic Dysfunction and Progression of Chronic Kidney Disease: Analysis of KNOW-CKD Data. J Am Heart Assoc 11, e025554, doi:10.1161/jaha.122.025554 (2022).

11 Suh, S. H. et al. Association of Left Ventricular Diastolic Dysfunction With Cardiovascular Outcomes in Patients With Pre-dialysis Chronic Kidney Disease: Findings From KNOW-CKD Study. Front Cardiovasc Med 9, 844312, doi:10.3389/fcvm.2022.844312 (2022).
